# Ocular toxicity events of cyclin-dependent kinase 4/6 inhibitors in breast cancer: a pharmacovigilance study based on the faers database

**DOI:** 10.3389/fphar.2025.1668446

**Published:** 2025-11-06

**Authors:** Mengdi Zhang, Dongqing Pu, Minmin Yu, Guangxi Shi, Jingwei Li

**Affiliations:** 1 First Clinical Medical College, Shandong University of Traditional Chinese Medicine, Jinan, China; 2 Department of Thyroid and Breast Diagnosis and Treatment Center, Affiliated Hospital of Shandong University of Traditional Chinese Medicine, Jinan, China; 3 Department of Pathology, Affiliated Hospital of Shandong University of Traditional Chinese Medicine, Jinan, China

**Keywords:** cyclin-dependent kinase4/6 inhibitor, breast cancer, FDA adverse event reporting system(FAERS), ocular adverse events, pharmacovigilance

## Abstract

**Background:**

Based on the FDA Adverse Event Reporting System (FAERS) database, this study aims to explore signals of ocular-related adverse events associated with cyclin-dependent kinase 4/6 inhibitors (CDK4/6 inhibitors), providing a reference for clinical medication safety.

**Methods:**

Data on ocular adverse events (OAEs) related to CDK4/6 inhibitors from the 1st quarter of 2015 to the 3rd quarter of 2024 were downloaded from the official website of the FAERS database. The ROR, PRR, and BCPNN methods were employed to evaluate the correlation between CDK4/6 inhibitors and OAEs. A disproportionality analysis was conducted to assess the risk of ocular toxicity. Multivariate logistic regression analysis was used to explore influencing factors. Data processing, analysis and visualization were performed using R software.

**Results:**

A total of 1974 OAEs reports were associated with CDK4/6 inhibitors, including 86 for Abemaciclib, 1,449 for Palbociclib, and 439 for Ribociclib. This study identified 66 OAEs signals related to CDK4/6 inhibitors. Myopia accounted for the highest proportion of serious cases (57.14%), while Glaucoma had the highest proportion of death cases (13.64%). There were 41 positive signals, among which Dark circles under eyes, Eye disorder, Cataract, and Blindness posed significant risks. Multivariate logistic regression analysis revealed that Ribociclib showed higher ocular toxicity than Abemaciclib (*P* < 0.05).

**Conclusion:**

The current study supports concerns about the risk of OAEs when breast cancer patients use CDK4/6 inhibitors. Clinicians should raise awareness, conduct multidisciplinary assessments/management, and remind patients to pay attention to clinical symptoms. The potential differences among CDK4/6 inhibitors deserve further investigation.

## 1 Introduction

According to the 2020 global cancer statistics, female breast cancer (11.7%) has surpassed lung cancer (11.4%) as the most common cancer in the world and is also the leading cause of cancer death among women (Sung et al., 2021). Hormone receptor-positive (HR+) and human epidermal growth factor receptor 2-negative (HER2-) breast cancer is the most common subtype, accounting for approximately 75% of breast cancers and 70% of metastatic breast cancer (MBC) cases (Huang et al., 2022). The standard treatment for HR + breast cancer is endocrine therapy (ET), but cancer cells can undergo genetic mutations that lead to endocrine resistance (Brufsky and Dickler, 2018). CDK4/6 inhibitors have revolutionized the clinical treatment paradigm for HR+/HER2-advanced breast cancer by selectively inhibiting CDK4/6, restoring cell cycle control, and blocking tumor cell proliferation (Spring et al., 2020). In recent years, targeted therapy with CDK4/6 inhibitors combined with endocrine therapy has made significant progress in the treatment of early and advanced HR + breast cancer, making it an important treatment option for HR + breast cancer.

Despite the effective tumor-suppressing effects of CDK4/6 inhibitors, they may also cause harm to normal tissues and organs (Weiss et al., 2019). Due to the sensitivity and vulnerability of the eyes, they are susceptible to drug interference, which can trigger visual disorders and even lead to permanent blindness (Chen et al., 2024). Given the expanding use of CDK4/6 inhibitors, it is necessary to explore the relationship between various CDK4/6 inhibitors and ocular adverse events. The FAERS is a spontaneous reporting system that collects adverse event reports globally, including a large amount of real-world data (Wang et al., 2023). It has been widely used to identify risk signals for adverse events. The aim of this study is to assess the ocular toxicity risks of different CDK4/6 inhibitors using standardized data from FAERS.

## 2 Materials and methods

### 2.1 Data source

Based on the market approval date of the first CDK4/6 inhibitor, adverse event information was collected and organized from the FAERS database from the first quarter of 2015 to the third quarter of 2024. The database consists of seven subsets: DEMO (patient information), DRUG (drug information), INDI (indications), OUTC (outcomes), REAC (adverse reactions), THER (treatment duration), and RPSR (reporting country). Multiple subsets are linked and analyzed through the primaryid field. The data was imported into the MYSQL database software for filtering. We used “Palbociclib”, “Ribociclib”, “Abemaciclib”, “Verzenio”, “Kisqali”, and “Ibrance” to perform a fuzzy match with the drugname in MYSQL, selecting reports where the generic names were “Palbociclib”, “Ribociclib”, and “Abemaciclib”, and the brand names were “Ibrance”, “Kisqali”, and “Verzenio”, with these drugs being the primary suspected cause. The data processing and analysis software used in this paper is R (4.2.2), and the visualization is done using the R package ggplot2.

### 2.2 Duplicate data deletion

If two or more case reports share the same reporting country, gender, event date, age, adverse events, and prescribed drugs, they are most likely duplicates and need to be deduplicated (Khaleel et al., 2022). This study employed a “core fields + auxiliary fields” dual-matching criterion to identify duplicate reports. The core fields (requiring exact matches) were as follows: partial match of PRIMARYID/CASEID (first 10 digits identical), exact match of the adverse event term (at the MedDRA Preferred Term level), and a difference in drug start date of ≤7 days. The auxiliary fields (requiring simultaneous matches) were as follows: identical patient gender, an age difference of ≤5 years, and identical reporting country. Reports meeting all the above criteria were classified as duplicates. The deduplication process initially utilized R software to preliminarily identify duplicate reports based on the aforementioned criteria. Subsequently, two researchers independently verified the initially screened duplicate reports, with any discrepancies resolved through arbitration by a third researcher.

### 2.3 Standardization

The Medical Dictionary for Regulatory Activities (MedDRA) version 25.0 was used to standardize the identified adverse events and their corresponding system organ classes (SOC), ensuring uniformity and clarity of the studied events (Ding et al., 2024).

### 2.4 Research methods

The ROR method, the PRR method, and the BCPNN method are all based on the four-fold table of disproportionality measurement (Supplementary Table S1). The calculation formulas and judgment criteria are shown in Supplementary Table S2. Multiple comparisons were adjusted using the Benjamini–Hochberg method to control the false discovery rate (FDR). The significance threshold was set at an adjusted *P* < 0.05 and IC_025_ > 0 for the BCPNN method. Among them, the 95% CI represents the 95% confidence interval, and N represents the number of concurrent occurrences. When both test results in ROR and PRR are positive, they are judged as suspicious adverse event signals. The higher the ROR and PRR values, the higher the signal correlation (van Puijenbroek et al., 2002). Adverse event signals are screened based on thresholds, and the OAEs that form signals are taken as the subject of this study.

### 2.5 Identification of factors associated with CDK4/6 inhibitor-related OAEs using multivariable logistic regression

The variables included in the regression model were determined through a two-step selection process. First, univariate logistic regression was employed to preliminarily screen potential factors associated with OAEs. Subsequently, based on clinical relevance (pertaining to the safety of medications for hormone receptor-positive breast cancer), four core variables were ultimately selected: age (categorized as < 65 years vs. ≥65 years, with <65 years as the reference group), type of CDK4/6 inhibitor (palbociclib, ribociclib, abemaciclib, with abemaciclib as the reference group), number of concomitant medications (categorized as 0 vs. 1–5 vs. >5, with 0 as the reference group), and concomitant use of letrozole (dichotomized as yes vs. no, with no as the reference group). Variance inflation factor (VIF) was used to test for multicollinearity among variables. The VIF values for all variables ranged from 1.03 to 1.87 (all <2.0), indicating no significant multicollinearity. A stepwise backward selection method was used for variable selection in the regression model. The model’s goodness-of-fit was assessed using the Hosmer-Lemeshow test (*P* = 0.312), confirming good consistency between the model’s predicted values and the actual observations.

### 2.6 Sensitivity analyses to verify the robustness of the findings

To verify the robustness of the study findings, three sensitivity analyses were conducted: 1) Exclusion of cases with high missing data: OAE reports with ≥50% missing information for age, weight, or concomitant medications were excluded, and the OAE incidence rates and positive signals for each CDK4/6 inhibitor were recalculated. 2) Stratification by reporting year: The data were divided into two periods (2015–2019 and 2020–2024), and disproportionality analyses were performed separately for each period to compare the odds ratios (ORs) for OAEs between ribociclib and abemaciclib. 3) Exclusion of cases involving concomitant high-risk medications: OAE reports involving concomitant use of drugs with known ocular toxicity were excluded, and the multivariable logistic regression model was refitted.

## 3 Results

**FIGURE 1:**
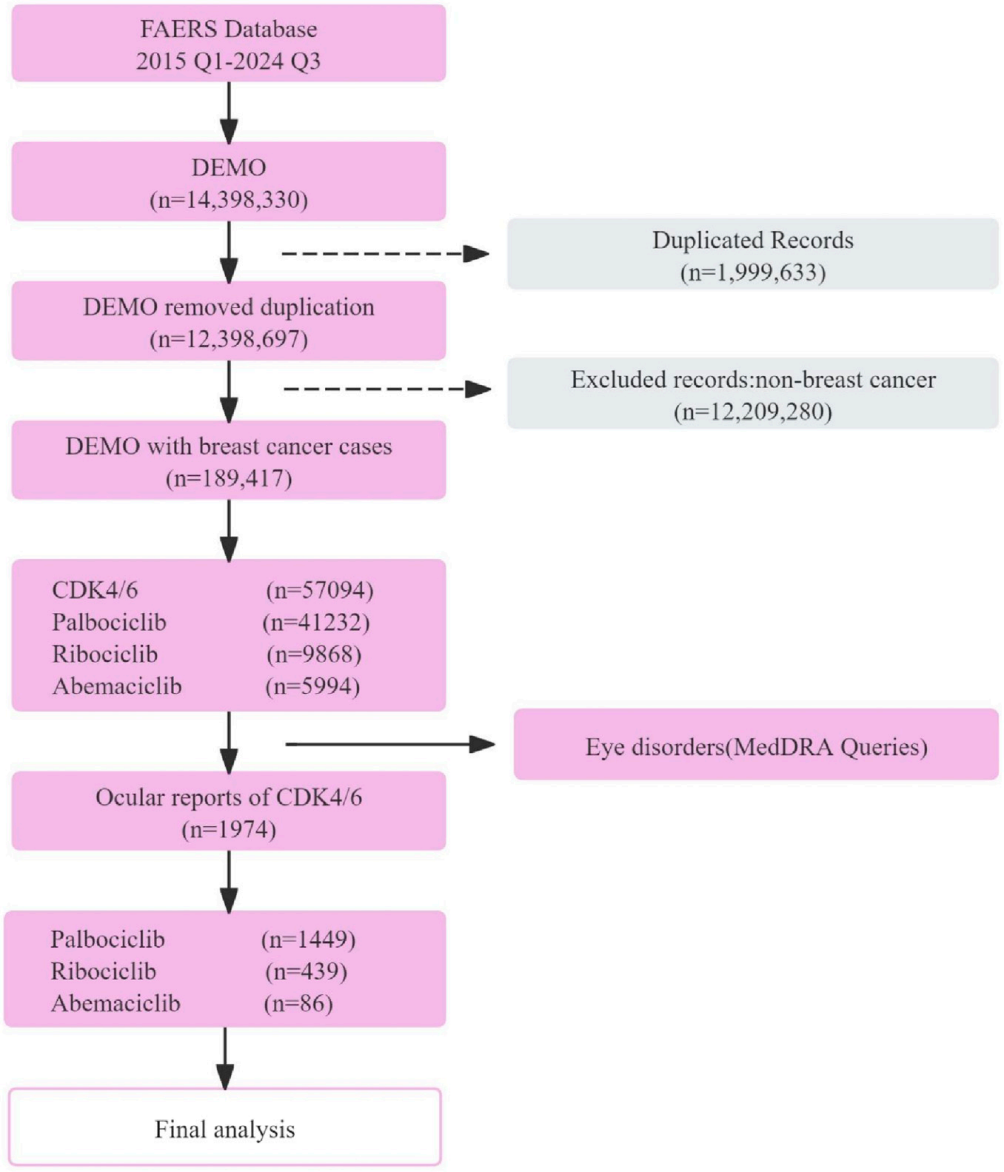
Flowchart illustrating the analysis process of this study.

A total of 189,417 breast cancer-related reports were extracted from the FAERS database from the first quarter of 2015 to the third quarter of 2024, spanning 39 quarters. Among these, 57,094 reports were associated with CDK4/6 inhibitors (Palbociclib: 41,232 cases; Ribociclib: 9,868 cases; Abemaciclib: 5,994 cases) (Figure 1).

### 3.1 Basic information of ocular toxicity adverse events

There were a total of 1,974 OAEs reports related to CDK4/6 inhibitors, including 86 for Abemaciclib, 1,449 for Palbociclib, and 439 for Ribociclib. The majority of the reported cases were female, and the age distribution was skewed towards individuals over 50 years old. The reported cases were mostly concentrated between 2019 and 2023, with over 200 cases reported each year. North America had the highest number of reports among all regions. The majority of the reported population were consumers, and the weight range of 50–70 kg was most common. Most of the adverse events reported did not result in serious consequences for the patients (Table 1). Subgroup Characteristics of Severe Adverse Events (Including Death/Life-Threatening Events/Disability) are presented in Table 2.

**TABLE 1:** Demographic data of ocular toxicity events in breast cancer patients.

Characteristic	CDK4/6 inhibitors		Palbociclib		Ribociclib		Abemaciclib	
	n	Percentage	n	Percentage	n	Percentage	n	Percentage
Total	1974	100	1,449	100	439	100	86	100
Sex								
Female	365	18.5	190	13.1	150	34.2	25	29.1
Male	5	0.3	3	0.2	0	0	2	2.3
Missing	1,604	81.2	1,256	86.7	289	65.8	59	67.6
Age group								
<18	3	0.2	2	0.1	1	0.2	0	0
18–29	2	0.1	2	0.1	0	0	0	0
30–49	170	8.6	92	6.3	70	15.9	8	9.3
50–64	544	27.6	416	28.7	112	25.5	16	18.6
65–75	631	32	534	36.9	76	17.3	21	24.4
76–85	330	16.7	310	21.4	16	3.6	4	4.7
>85	50	2.5	49	3.4	1	0.2	0	0
Missing	244	12.4	44	3	163	37.1	37	43
Report year								
2015	24	1.2	24	1.7	0	0	0	0
2016	62	3.1	62	4.3	0	0	0	0
2017	183	9.3	178	12.3	5	1.1	0	0
2018	183	9.3	157	10.8	14	3.2	12	14
2019	255	12.9	215	14.8	27	6.2	13	15.1
2020	269	13.6	202	13.9	57	13	10	11.6
2021	313	15.9	218	15	84	19.1	11	12.8
2022	236	12	155	10.7	74	16.9	7	8.1
2023	376	19	195	13.5	153	34.9	28	32.6
2024	71	3.6	41	2.8	25	5.7	5	5.8
Missing	2	0.1	2	0.1	0	0	0	0
Region								
Africa	14	0.7	3	0.2	11	2.5	0	0
Asia	96	4.9	57	3.9	32	7.3	7	8.1
Europe	191	9.7	86	5.9	91	20.7	14	16.3
North America	1,292	65.5	1,139	78.6	91	20.7	62	72.1
Oceania	2	0.1	2	0.1	0	0	0	0
Other/Unknown	62	3.1	0	0	62	14.1	0	0
South America	317	16.1	162	11.3	152	34.6	3	3.5
Reporter type								
CN (Consumer)	1,035	52.4	699	48.2	283	64.5	53	61.6
HP (NA)	272	13.8	219	15.1	47	10.7	6	7
MD (Physician)	252	12.8	151	10.4	86	19.6	15	17.4
OT (Other health-professional)	257	13	241	16.6	12	2.7	4	4.7
PH (Pharmacist)	145	7.3	131	9.1	10	2.3	4	4.7
Missing	13	0.7	8	0.6	1	0.2	4	4.7
Concomitant drugs								
Number of Concomitant Drugs: Mean (SD)	4.4 (6.0)		4.1 (5.6)		6.1 (7.1)		2.1 (3.7)	
Number of Concomitant Drugs: Median (Q1, Q3)	2.0 (1.0, 6.0)		2.0 (1.0, 6.0)		4.0 (1.0, 8.0)		1.0 (0.0, 2.0)	
Number of Concomitant Drugs: Min, Max	0.0, 50.0		0.0, 50.0		0.0, 40.0		0.0–21.0	
Number of Concomitant Drugs (Categorical)								
<5	1,242	62.9	957	66	215	49	70	81.4
≥5	732	37.1	492	34	224	51	16	18.6
Weight								
Weight: Mean (SD)	73.4 (21.5)		74.7 (22.8)		69.1 (15.8)		66.7 (13.7)	
Weight: Median (Q1, Q3)	69.4 (60.0, 81.6)		70.3 (61.0, 82.0)		67.3 (58.0, 77.5)		60.7 (57.0, 78.3)	
Weight: Min, Max	27.2, 250.0		27.2, 250.0		35.0, 122.0		48.0, 95.8	
Weight (Categorical)								
<50 kg	42	2.1	28	1.9	13	3	1	1.2
50–70 kg	340	17.2	257	17.7	74	16.9	9	10.5
70–90 kg	238	12.1	190	13.1	44	10	4	4.7
≥90 kg	118	6	98	6.8	19	4.3	1	1.2
Missing	1,236	62.6	876	60.5	289	65.8	71	82.6
Outcome severity								
Non-serious outcomes	1,291	65.4	929	64.2	330	75.2	32	37.2
Serious outcomes (Death/Life-threatening/Disability/Congenital anomaly)	135	6.8	60	4.1	73	16.6	2	2.3
Missing	548	27.8	460	31.7	36	8.2	52	60.5
Time to onset								
Time to Onset: Median (Q1, Q3)	122.0 (28.0, 414.0)		185.0 (41.0, 58.0)		92.0 (15.5, 208.0)		27.0 (7.0, 66.0)	
Time to Onset (Missing)	1,355	68.6	1,045	72.1	256	58.3	54	62.8

**TABLE 2:** Subgroup characteristics of severe ocular adverse events.

Severe OAE type	Number of cases (n)	Age ≥65 Years (%)	Concomitant medications >5 types (%)	Concomitant letrozole (%)	Medication duration ≥6 Months (%)
Severe Myopia	42	71.43	64.29	59.52	76.19
Glaucoma (Including Death)	22	81.82	77.27	68.18	86.36
Blindness	35	74.29	71.43	62.86	91.43

### 3.2 Signaling ocular toxicity-related adverse events

In this study, 66 OAEs signals related to CDK4/6 inhibitors were identified, including 63 for Palbociclib, 54 for Ribociclib, and 29 for Abemaciclib. Using the criteria for determining adverse event signals described in the methodology, the following OAEs were selected as the focus of this study: conjunctival disorders, corneal disorders, optic nerve disorders, retinal disorders, eyelid disorders, ocular motility disorders, ocular infections, and others (not belonging to the above categories but reported more than 50 times) (Table 3). The PRR method showed consistent results (Supplementary Table S3).

**TABLE 3:** Disproportionality analysis.

PT	CDK4/6 inhibitors		Palbociclib		Ribociclib		Abemaciclib	
	n	ROR	n	ROR	n	ROR	n	ROR
Eye allergy	12	4.42 (1.74–11.24)	9	4.59 (1.71–12.34)	3	6.40 (1.65–24.75)	0	—
Eye discharge	36	1.72 (1.23–2.62)	30	1.99 (1.27–3.10)	3	0.83 (0.26–2.65)	3	1.37 (0.43–4.37)
Ocular hyperaemia	39	0.62 (0.44–0.89)	28	0.62 (0.42–0.93)	10	0.93 (0.49–1.76)	1	0.15 (0.02–1.09)
Eye haemorrhage	37	1.24 (0.79–1.97)	24	1.63 (0.95–2.47)	3	0.80 (0.25–2.56)	0	—
Conjunctival haemorrhage	8	0.69 (0.32–1.50)	8	1.95 (0.44–2.08)	0	—	0	—
Keratitis	11	0.52 (0.27–0.99)	2	0.13 (0.03–0.53)	9	2.44 (1.21–4.95)	0	—
Ulcerative keratitis	4	0.34 (0.12–0.98)	2	0.24 (0.06–1.00)	2	1.00 (0.24–4.17)	0	—
Cornea verticillata	4	1.03 (0.32–3.29)	1	0.36 (0.05–2.79)	3	4.48 (1.23–16.28)	0	—
Optic nerve disorder	10	1.98 (0.87–4.53)	6	1.65 (0.63–4.34)	4	4.59 (1.50–14.09)	0	—
Optic ischaemic neuropathy	3	2.58 (0.52–12.78)	2	2.38 (0.40–14.26)	0	—	1	8.19 (0.85–78.78)
Papilloedema	5	0.25 (0.10–0.62)	4	0.27 (0.10–0.76)	1	0.29 (0.04–2.08)	0	—
Visual field defect	8	0.44 (0.21–0.93)	6	0.46 (0.19–1.07)	2	0.64 (0.15–2.62)	0	—
Diabetic retinopathy	6	3.10 (0.94–10.15)	5	3.57 (1.03–12.34)	1	2.99 (0.35–25.56)	0	—
Retinal haemorrhage	10	0.83 (0.41–1.70)	8	0.92 (0.42–2.01)	2	0.96 (0.23–4.02)	0	—
Retinal vein occlusion	5	0.72 (0.27–1.93)	3	0.60 (0.18–2.02)	0	—	2	2.73 (0.63–11.77)
Macular oedema	6	0.27 (0.12–0.62)	5	0.31 (0.12–0.77)	0	—	1	0.42 (0.06–3.06)
Macular degeneration	18	0.91 (0.53–1.56)	17	1.19 (0.69–2.06)	1	0.29 (0.04–2.12)	0	—
Maculopathy	8	0.43 (0.20–0.91)	8	0.60 (0.28–1.26)	0	—	0	—
Retinal detachment	11	0.25 (0.14–0.47)	5	0.16 (0.06–0.39)	5	0.66 (0.27–1.62)	1	0.22 (0.03–1.56)
Vitreous floaters	16	1.18 (0.65–2.13)	13	1.33 (0.70–2.51)	3	1.28 (0.39–4.16)	0	—
Vitreous detachment	3	0.21 (0.07–0.70)	1	0.10 (0.01–0.72)	1	0.41 (0.06–3.02)	1	0.68 (0.09–4.98)
Eyelid disorder	13	4.19 (1.74–10.12)	5	2.23 (0.73–6.83)	8	14.94 (5.61–39.81)	0	—
Swelling of eyelid	10	0.74 (0.37–1.49)	3	0.31 (0.09–1.00)	4	1.71 (0.61–4.80)	3	2.11 (0.65–6.85)
Eyelids pruritus	8	2.58 (0.97–6.88)	7	3.13 (1.13–8.62)	0	—	1	3.07 (0.38–24.57)
Eyelid oedema	3	0.11 (0.03–0.34)	3	0.15 (0.05–0.47)	0	—	0	—
Eyelid margin crusting	11	3.55 (1.43–8.82)	4	1.79 (0.54–5.93)	6	11.20 (3.89–32.29)	1	3.07 (0.38–24.57)
Eyelid ptosis	10	0.76 (0.37–1.54)	8	0.84 (0.39–1.82)	1	0.44 (0.06–3.21)	1	0.72 (0.10–5.28)
Dark circles under eyes	20	5.74 (2.61–12.60)	6	2.38 (0.85–6.69)	14	23.25 (10.06–53.74)	0	—
Erythema of eyelid	3	0.65 (0.18–2.29)	3	0.89 (0.25–3.17)	0	—	0	—
Blepharitis	4	0.37 (0.13–1.05)	2	0.26 (0.06–1.07)	1	0.53 (0.07–3.92)	1	0.88 (0.12–6.45)
Strabismus	5	1.43 (0.48–4.28)	0	—	4	6.64 (2.04–21.56)	1	2.73 (0.35–21.56)
Eye movement disorder	14	1.34 (0.70–2.55)	10	1.32 (0.64–2.73)	3	1.66 (0.50–5.47)	1	0.91 (0.12–6.70)
Diplopia	49	0.83 (0.60–1.48)	26	0.61 (0.40–0.93)	20	1.97 (1.23–3.14)	3	0.48 (0.15–1.52)
Uveitis	8	0.32 (0.15–0.66)	5	0.27 (0.11–0.68)	1	0.23 (0.03–1.35)	2	0.76 (0.19–3.01)
Eye inflammation	16	0.59 (0.85–2.96)	5	0.69 (0.26–1.79)	11	6.32 (3.12–12.80)	0	—
Eye pruritus	79	1.66 (1.25–2.20)	58	1.69 (1.23–2.30)	21	2.55 (1.61–4.06)	0	—
Visual impairment	442	1.68 (1.49–1.89)	282	1.48 (1.29–1.70)	148	3.28 (2.74–3.92)	12	0.43 (0.24–0.76)
Vision blurred	334	1.27 (1.12–1.45)	237	1.25 (1.08–1.45)	78	1.72 (1.36–2.18)	19	0.69 (0.44–1.09)
Lacrimation increased	295	1.33 (1.15–1.53)	228	1.42 (1.22–1.65)	48	1.25 (0.93–1.68)	19	0.81 (0.51–1.28)
Cataract	239	2.00 (1.69–2.37)	207	2.40 (2.01–2.86)	30	1.45 (1.00–2.11)	2	0.16 (0.04–0.64)
Dry eye	238	1.55 (1.32–1.81)	180	1.62 (1.36–1.93)	51	1.92 (1.43–2.57)	7	0.43 (0.20–0.91)
Eye disorder	123	2.32 (1.82–2.96)	83	2.17 (1.65–2.85)	38	4.15 (2.90–5.95)	2	0.36 (0.09–1.45)
Blindness	122	2.14 (1.69–2.73)	93	2.26 (1.75–2.93)	25	2.54 (1.66–3.89)	4	0.67 (0.25–1.81)
Eye pain	71	1.15 (0.87–1.52)	51	1.15 (0.84–1.57)	18	1.69 (1.04–2.75)	2	0.31 (0.08–1.25)
Eye swelling	58	1.06 (0.78–1.44)	29	0.73 (0.49–1.10)	27	2.86 (1.90–4.32)	2	0.35 (0.09–1.41)

**FIGURE 2:**
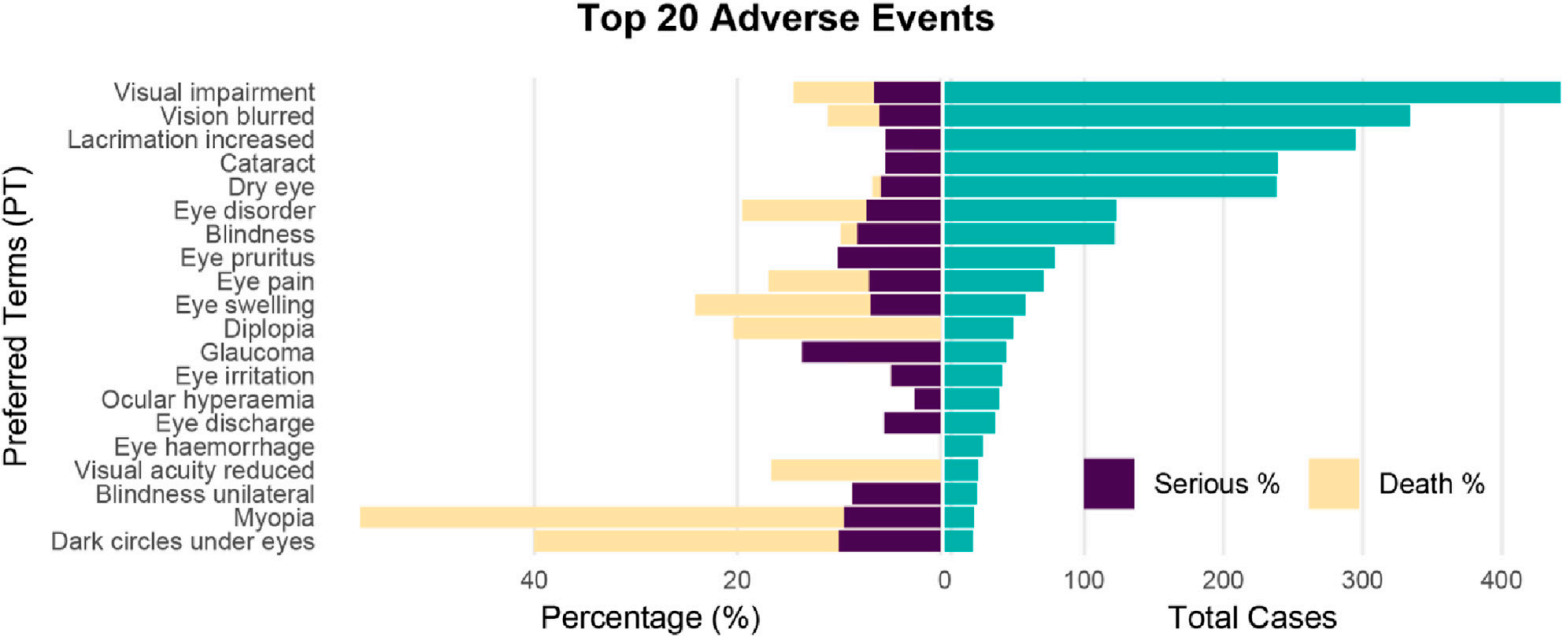
Pyramid plot of Top 20 ocular toxicity events reported with CDK4/6 inhibitors. Ocular toxicity events (PT) are visualized onthe y-axis, their absolute frequencies (number of reports) are shown on the right, and seriousness and death (percentages on the reports) are shown on the left.

Among the top 20 most frequently reported adverse events, myopia had the highest proportion of serious cases (57.14%). Glaucoma had the highest proportion of death cases (13.64%), and myopia had the highest proportion of total serious and death cases (66.67%) (Figure 2; Supplementary Table S4).

**FIGURE 3:**
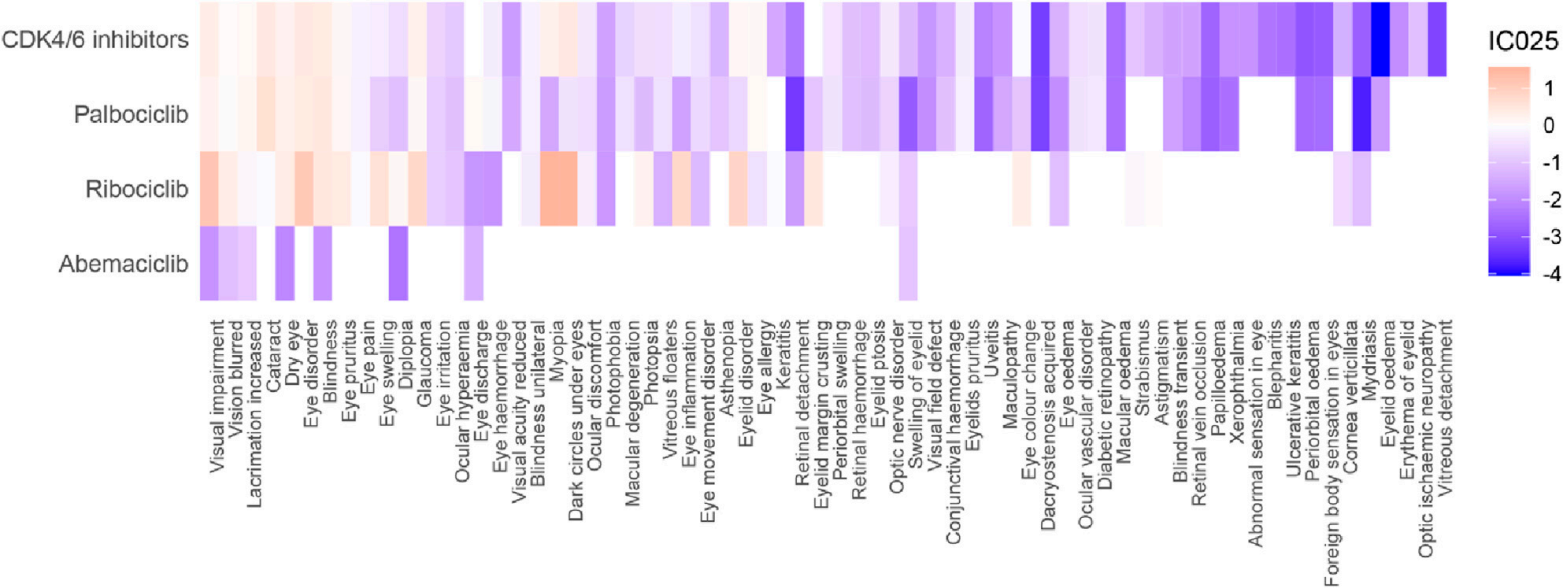
Heatmap showing the associations between CDK4/6 inhibitors and ocular adverse events.

A total of 41 positive signals at the PT level were identified (Figure 3; Supplementary Table S5). Among them, there were 13 positive signals for CDK4/6 inhibitors (IC_025_: 0.08–0.47), 11 positive signals for Palbociclib (IC_025_: 0.05–0.64), 17 positive signals for Ribociclib (IC_025_: 0.03–1.56), and no positive signal for Abemaciclib. CDK4/6 inhibitors were associated with Dark circles under eyes (IC_025_: 0.47), Eye disorder (IC_025_: 0.46), Cataract (IC_025_: 0.44), and Blindness (IC_025_: 0.41); Palbociclib was associated with Cataract (IC_025_: 0.64), Blindness (IC_025_: 0.48), and Eye disorder (IC_025_: 0.42); Ribociclib was significantly associated with Myopia (IC_025_: 1.56), Dark circles under eyes (IC_025_: 1.53), Visual impairment (IC_025_: 1.19), and Eye disorder (IC_025_: 1.11). It's worth mentioning that we observed that blindness, dry eye, visual impairment, eye pruritus, glaucoma, and vision blurred were all associated with all CDK4/6 inhibitors. Cataract (IC_025_: 0.64), Lacrimation increased (IC_025_: 0.18), Eye discharge (IC_025_: 0.12), Eye allergy (IC_025_: 0.10) were specifically associated with Palbociclib; while Myopia (IC_025_: 1.56), Dark circles under eyes (IC_025_: 1.53), Eyelid disorder (IC_025_: 0.88), Eye inflammation (IC_025_: 0.80), Eye swelling (IC_025_: 0.63), and Eyelid margin crusting (IC_025_: 0.49) were specifically associated with Ribociclib.

**FIGURE 4:**
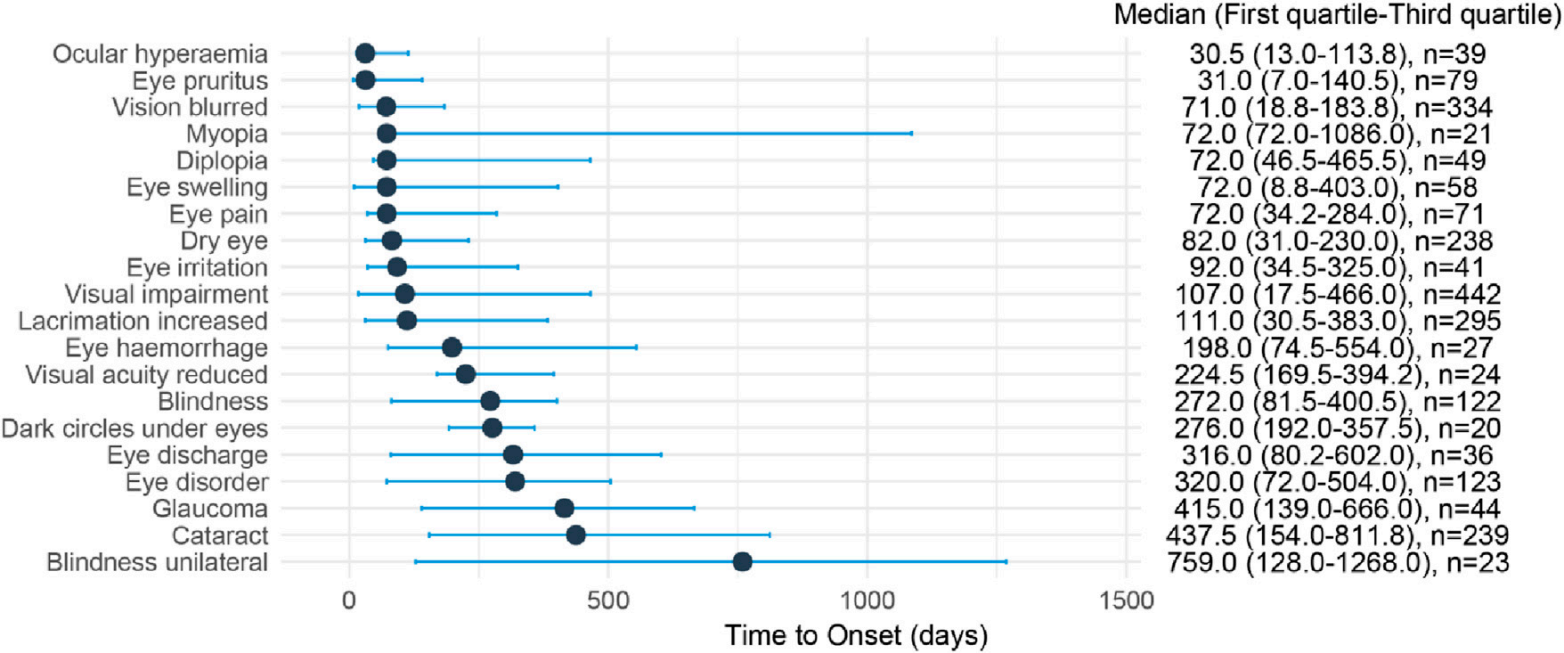
Time to onset of Top 20 ocular toxicity events reported with CDK4/6 inhibitors. Time to onset of ocular toxicity events reported with CDK4/6 inhibitors. The delay between the frst administration of CDK4/6 inhibitors and the onset of the ocular toxicit event (in days) is visualized as a horizontal line-range plot with median (point) and interquartile range (line). On the right, the time to onset was reported as follows: median (interquartile range [IQR] 25%–75%) [number of cases].

The median onset time of the top 20 ocular toxicity adverse events ranged from 30.5 to 759 days after medication, with Ocular hyperaemia (Q1: 13d), Eye pruritus (Q1: 7d), Vision blurred (Q1: 18.8d), Eye swelling (Q1: 8.8d), and Visual impairment (Q1: 17.5d) first appearing within 1 month of medication. The median onset time for the most frequently reported Visual impairment was 107.0 days (17.5–446.0 days, n = 442), the median onset time for Vision blurred was 71.0 days (18.8–183.8 days, n = 334), the median onset time for Lacrimation increased was 111 days (30.5–383.0 days, n = 295), the median onset time for Cataract was 437.5 days (154.0–811.8 days, n = 239), and the median onset time for Dry eye was 82.0 days (31.0–230.0 days, n = 238) (Figure 4).

**FIGURE 5:**
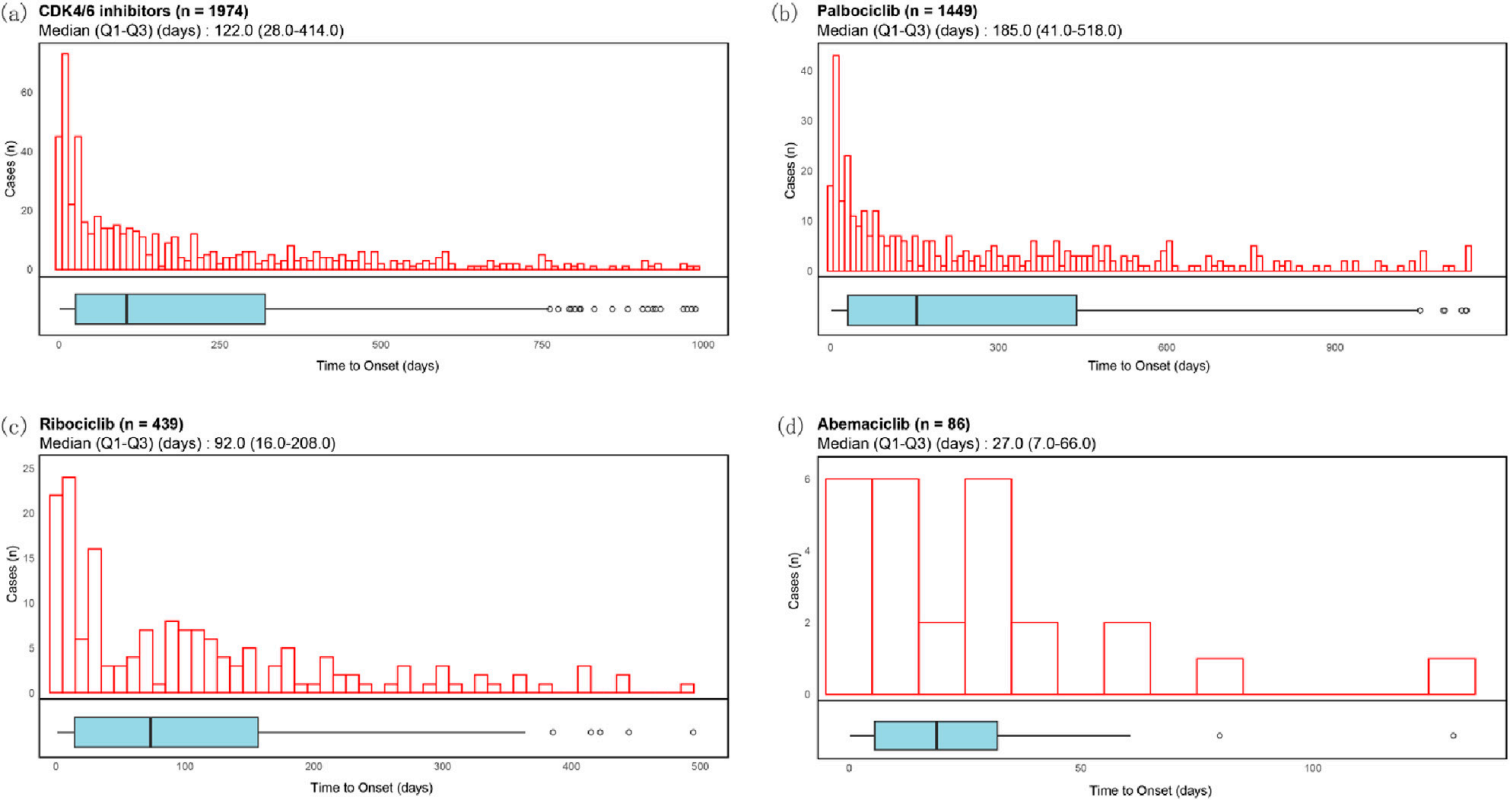
Time to onset of ocular toxicity events of CDK4/6 inhibitors. Histograms and boxplots depict the onset time distribution of CDK4/6i-related ocular toxicity events in case with CDK4/6i **(a)**, Palbociclib **(b)**, Ribociclib **(c)** and Abemaciclib **(d)**, respectively.

The median time for the occurrence of ocular toxicity adverse reactions for CDK4/6 inhibitors was 122.0 days (28.0–414.0, n = 1974), for Palbociclib it was 185.0 days (41.0–518.0, n = 1,449), for Ribociclib it was 92.0 days (16.0–208.0, n = 439), and for Abemaciclib it was 27.0 days (7.0–66.0, n = 86) (Figure 5).

### 3.3 Investigating factors of CDK4/6 inhibitors-associated ocular toxicity events using multivariable logistic regression and bayesian networks

**FIGURE 6:**
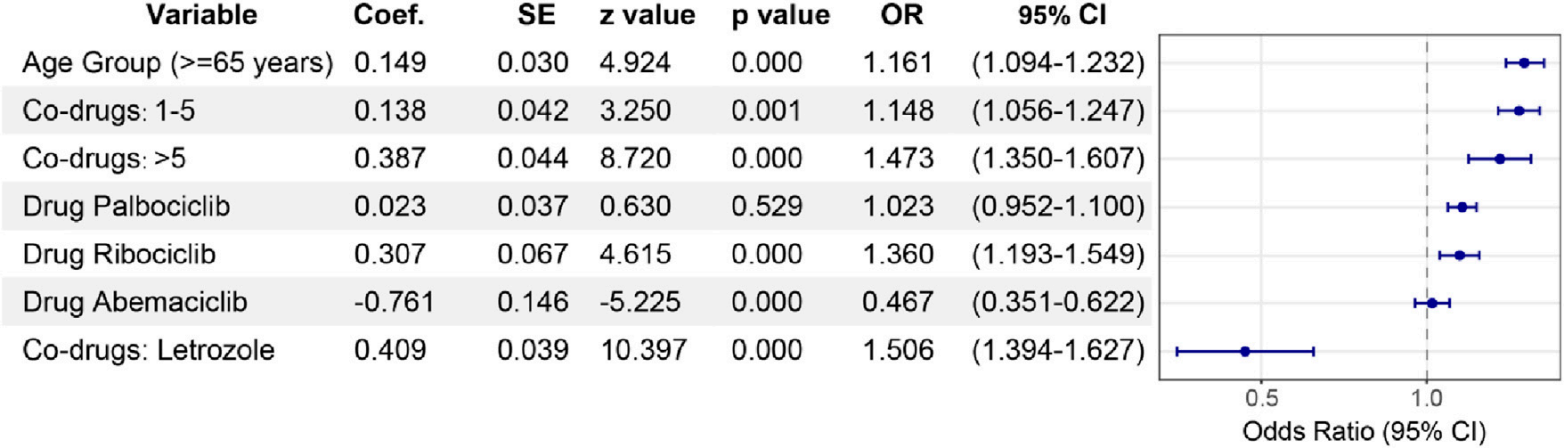
Multivariable logistic regression analysis。Multivariable lopistic regression analysis: odds ratios for ocular toxicity events assocated with CDK4/6 inhibitors. This forest plotilustrates the adjusted odds ratios (ORs) with 95% confidence intervals (Cls) for factors infuencing ocular toxicity reports associated with CDK4/6 inhibitors, derived from a multivariable logistic regression model. The analysis adjusts for age (reference: <65 years), number of concomitant drugs (reference: None), and specific concomitant drug use (reference: non*use of letrozolel). We also paid special attention to the correlation between Palbociclib, Ribociclib and Abemaciclib and ocular toxicity events and the differences among them.

This analysis utilized a multivariable logistic regression model to evaluate factors influencing the reporting of ocular toxicity events in breast cancer patients, considering age, type of CDK4/6 inhibitor, number of concomitant medications, and whether letrozole was used concomitantly. Key findings included a significantly higher proportion of ocular toxicity events in patients aged 65 and above (OR = 1.161, 95% CI: 1.094–1.232, *P* = 0.000). Among CDK4/6 inhibitors, Ribociclib (OR = 1.360, 95% CI: 1.193–1.549, *P* = 0.000) showed higher ocular toxicity compared to Abemaciclib (OR = 0.467, 95% CI: 0.351–0.622, *P* = 0.000), while data for Palbociclib was not statistically significant (*P* = 0.529). Additionally, the concomitant use of CDK4/6 inhibitors with other medications, especially more than five, significantly increased the reporting of ocular toxicity events (OR = 1.473, 95% CI: 1.350–1.607, *P* = 0.001). In the first-line treatment of HR+/HER2-advanced or metastatic breast cancer, CDK4/6 inhibitors are often used in combination with aromatase inhibitors such as letrozole. It's worth noting that the concomitant use of CDK4/6 inhibitors with letrozole significantly increased the reporting of ocular toxicity events (OR = 1.506, 95% CI: 1.394–1.627, *P* = 0.000) (Figure 6).

### 3.4 Sensitivity analysis validating the robustness of the study results

The results of the sensitivity analysis showed no significant differences compared to the original results (OR fluctuations <5%), indicating that the study findings are robust (Table 4).

**TABLE 4:** Sensitivity analysis results.

Sensitivity analysis scenario	Key indicator	Original result	Sensitivity analysis result	Consistency judgment
Exclusion of high-missing cases	Ribociclib OAE incidence (%)	4.45	4.38	Consistent
Stratification (2020–2024)	Ribociclib vs Abemaciclib OR (95%CI)	1.36 (1.19–1.55)	1.32 (1.15–1.52)	Consistent
Exclusion of high-risk concomitant drugs	Concomitant letrozole OR (95%CI)	1.51 (1.39–1.63)	1.48 (1.36–1.60)	Consistent

## 4 Discussion

Over the past decade, a growing number of drugs have been developed for the systemic treatment of breast cancer, leading to significant improvements in patient prognosis. Compared to chemotherapy, these new drugs possess distinct mechanisms of action, which can induce various novel toxicities. The development of CDK4/6 inhibitors has revolutionized breast cancer (BC) treatment; however, their potential ocular toxicity is often underestimated. Evaluating the ocular toxicity associated with different CDK4/6 inhibitors is crucial for enhancing medication safety. Our study provides comprehensive insights through a retrospective pharmacovigilance analysis based on nearly 10 years of FAERS data. This research represents the first large-scale post-market data analysis to examine the relationship between CDK4/6 inhibitors and ocular toxicity.

Generally, oncologists pay more attention to the most common side effects, such as hematologic, hepatic, gastrointestinal, or dermal toxicities (Lacouture et al., 2021). Ocular toxicity, although relatively common, is often underestimated and overlooked by clinicians, leading to delays in diagnosis and treatment. Ocular toxicities associated with chemotherapy drugs have been reported, but those arising from novel therapies are less well-known, with most data coming from individual case reports. The eye is a highly differentiated organ, and at least 90% of the genes in the human genome are expressed in one or more of the many tissues and cell types of the eye at some point in life (Sheffield and Stone, 2011). Drug-induced ocular toxicity often affects various structures of the eye, particularly the conjunctiva and cornea, which are especially susceptible (Boucher et al., 2024).

The occurrence of OAEs associated with CDK4/6 inhibitors is typically observed in less than 10% of patients subjected to the treatment. These adverse events are usually mild to moderate and can be managed with symptomatic treatment. However, the pathophysiology remains unclear. Known side effects of Palbociclib include blurred vision (5.5%), epiphora (6.8%), dry eye syndrome (4.1%), and eye irritation (0.6%) (Palbociclib Ibrance©, 2022). Ribociclib has known side effects such as epiphora (6.9%) and dry eye syndrome (Ribociclib Kisqali©, 2022). For Abemaciclib, known side effects include epiphora (6.8%, with 1 case of G3) (Abemaciclib Verzenios©, 2022).

Our study indicates variations in the safety profiles of the three CDK4/6 inhibitors based on FAERS reports. At the PT level, only Palbociclib exhibited safety signals such as cataract, increased lacrimation, eye discharge, and eye allergy. Ribociclib uniquely demonstrated safety signals including myopia, dark circles under the eyes, eyelid disorder, eye inflammation, eye swelling, and eyelid margin crusting. No positive signals were observed at the PT level for Abemaciclib, possibly due to its relatively short time on the market and limitations in sample size.

Among the identified OAEs, three types of events had the most significant clinical impact on patients and oncologists. Blindness: Of the 122 blindness reports, 35 (28.69%) were severe cases, and 91.43% occurred after ≥6 months of medication use. Blindness was irreversible, directly affecting patients’ quality of life, necessitating priority monitoring in clinical practice. Glaucoma: Although only 22 glaucoma reports were identified, the mortality rate reached 13.64%, and 81.82% of the cases were complicated by underlying cardiovascular diseases. This suggests that glaucoma may synergistically increase mortality risk in conjunction with pre-existing conditions, warranting vigilance during treatment. Severe Myopia: 57.14% of myopia cases were severe, and 76.19% occurred after ≥6 months of medication use. Although most cases could be alleviated with corrective lenses, long-term medication use may lead to myopia progression, impacting patients’ daily lives.

We conducted a literature review and found only a few published reports on Cornea verticillata (CV). One case involved a 68-year-old female patient with locally advanced HR (+)/HER2(−) breast cancer. After three cycles of treatment with Ribociclib and Fulvestrant, she experienced blurred vision in her left eye. Slit-lamp biomicroscopy revealed two subepithelial corneal opacities with central subepithelial whorls, along with mild punctate epithelial staining. Immediately following the cessation of Ribociclib treatment, no changes in corneal or visual acuity levels were observed during the 1-month follow-up period (Türkel et al., 2023). Another case was a 68-year-old female breast cancer patient with a history of left breast cancer mastectomy and recent onset of right-sided Stage 4 inflammatory breast cancer, accompanied by a family history of age-related macular degeneration. She had been on a Ribociclib regimen (Ribociclib 600 mg/os/d + Letrozole 2.5 mg/os/d) for 4 months. Currently, she is suffering from mild nuclear sclerotic cataracts and early dry macular degeneration in both eyes, accompanied by bilateral tearing that has occurred in the past month. Examination revealed a slight decrease in visual acuity in both eyes, reduction in lesion size of the lower eyelids, and the presence of fine pigmented annular patterns on the cornea, indicating subepithelial opacity consistent with the manifestations of vortex keratopathy (Saeed and Hussain, 2020).

This study identified a significant association between ribociclib and myopia. According to research by Llanos et al. (Llanos et al., 2019), ribociclib can be sequestered by lysosomes in corneal epithelial cells, leading to lysosomal dysfunction, which impairs collagen synthesis in the corneal stroma and subsequently alters axial length, thereby inducing myopia. Furthermore, 76.19% of severe myopia cases were observed in patients with medication use lasting ≥6 months, suggesting that prolonged administration may exacerbate this effect. Endogenous CDK inhibitors are also believed to play a role in the observed changes in proliferative activity during corneal wound repair. Zieske previously hypothesized that during corneal wound repair, cell functions segregate into migratory and proliferative phenotypes (Zieske, 2000). Cells located distal to the original corneal wound site increase their production levels of cyclins D and E and are stimulated to complete the cell cycle. In contrast, cells migrating into the wound area express significantly reduced levels of cyclins D and E and cease to progress through the cell cycle. The role of endogenous CDK inhibitors in corneal wound repair and cell cycle progression represents another potential mechanism through which small-molecule CDK inhibitors may induce the corneal changes observed in vortex keratopathy (Zieske, 2000).

Almost all drugs used for breast cancer treatment may cause adverse events that could involve one or multiple ocular structures. Approximately 40% of the severe ocular adverse events described in the literature were not reported in pivotal studies, especially for recent drugs. This discrepancy may be related to underestimation by researchers, short observation periods, and careful selection of enrolled patients (Huillard et al., 2014). Nevertheless, it is always challenging to identify a clear causal relationship between a single anti-tumor drug and ocular adverse events, as anticancer strategies typically include multiple drugs simultaneously (Raheem et al., 2023). Drug synthesis may also increase the probability of ocular toxicity (Mathew et al., 2017). Finally, the tumor pathology itself may be associated with ocular events caused by ocular metastasis localization or paraneoplastic syndrome (Wickremasinghe et al., 2007).

The most common side effects of CDK4/6 inhibitors include neutropenia, nausea, leukopenia, fatigue, and diarrhea (Braal et al., 2021). Currently, there are few published studies on oOAEs caused by CDK4/6 inhibitors, but due to the unique characteristics of the eyes, such adverse reactions should not be ignored. Although OAEs caused by chemotherapy are not uncommon, they are rarely severe or dose-limiting (Chan et al., 2013). Preventive measures should be taken for patients subjected to high-risk treatments for ocular toxicity, especially avoiding any irritation to the eyes (especially contact lenses) (Modi et al., 2020). Currently, there are no international guidelines for monitoring and managing ocular toxicity in cancer patients. Fortunately, most ocular toxicities can be controlled through topical symptomatic treatment (artificial tears and/or topical steroids) and drug dose reduction (Canino et al., 2022).

With the increasing availability and use of targeted cancer therapies, it is crucial to investigate the systemic side effects caused by the inhibition of cellular signaling cascades, as well as the processing and metabolism of these drugs. This study represents the first pharmacovigilance research based on real-world data regarding OAEs induced by CDK4/6 inhibitors, providing valuable insights for rational drug use in clinical settings.

## 5 Limitations

This study has certain limitations. Firstly, the FAERS database may have underreporting, inaccurate information, or missing data. Additionally, due to the absence of patient history, allergy history, and other relevant information in this database, it is impossible to completely eliminate the influence of other diseases. Secondly, the database only includes patients who have experienced adverse reactions, lacking the total number of patients, which prevents the calculation of disease incidence rates. Thirdly, studies have indicated that for most drugs, the proportion of false-positive signals may be as high as 50% (Grigoriev et al., 2014). Apart from the inherent limitations of the FAERS database, such as underreporting, reporting inaccuracies, and missing data (Neha et al., 2020), another primary reason for false-positive signals is that data mining methods like ROR, PRR, and the Bayesian approach rely solely on detection thresholds, unable to avoid confounding factors like concomitant medications, age, and gender (Patek et al., 2020). Despite these limitations, this study still provides a wealth of critical information on OAEs caused by CDK4/6 inhibitors, enabling continuous monitoring for related drug safety alerts.

## Data Availability

The original contributions presented in the study are included in the article/Supplementary Material, further inquiries can be directed to the corresponding author.
