# Supplementary material for: Ocular toxicity events of cyclin-dependent kinase 4/6 inhibitors in breast cancer: a pharmacovigilance study based on the faers database
Source: Front Pharmacol. 2025 Nov 6;16:1668446. doi: 10.3389/fphar.2025.1668446 (PMC12631214; doi:10.3389/fphar.2025.1668446)
Supplement: Supplementary file 1 [file Table1.docx]

**Table S1 Fourfold Table of Disproportionality Measures**

| Drug types | Number of target ADE reports | Number of other ADE reports | Total |
| --- | --- | --- | --- |
| Target drug | a | b | a+b |
| Other drugs | c | d | c+ |
| Total | a+c | b+d | N=a+b+c+d |
