# Supplementary material for: Ocular toxicity events of cyclin-dependent kinase 4/6 inhibitors in breast cancer: a pharmacovigilance study based on the faers database
Source: Front Pharmacol. 2025 Nov 6;16:1668446. doi: 10.3389/fphar.2025.1668446 (PMC12631214; doi:10.3389/fphar.2025.1668446)
Supplement: Supplementary file 2 [file Table2.docx]

**Table S2 Summary of Major Algorithms Used for Signal Detection**

| Algorithms | Equation | Criteria |
| --- | --- | --- |
| ROR | ROR=ad/bc | lower limit of 95% CI>1, N≥2 |
|  | 95%CI=e^ln(ROR)±1.96(1/a+1/b+1/c+1/d)^0.5^ |  |
| PRR | PRR=(a(c+d))/(c(a+b)) | PRR≥2, χ^2^≥4, N≥3 |
|  | χ^2^ = [(ad − bc)^2](a + b + c + d)/[(a + b)(c + d)(a + c)(b + d)] |  |
| BCPNN | IC=log_2_a(a+b+c+d)(a+c)(a+b) | IC_025_>0 |
|  | IC_025_=e^ln(IC)-1.96(1/a+1/b+1/c+1/d)^0.5^ |  |
|  | EBGM05=e^ln(EBGM)-1.64(1/a+1/b+1/c+1/d)^0.5^ |  |
