# Supplementary material for: Ocular toxicity events of cyclin-dependent kinase 4/6 inhibitors in breast cancer: a pharmacovigilance study based on the faers database
Source: Front Pharmacol. 2025 Nov 6;16:1668446. doi: 10.3389/fphar.2025.1668446 (PMC12631214; doi:10.3389/fphar.2025.1668446)
Supplement: Supplementary file 3 [file Table5.docx]

**Table S5 X^2^ and IC_025_ of Positive Signal of 41 PTs**

| PT | X^2^ | IC_025_ | Cases |
| --- | --- | --- | --- |
| **CDK4/6 inhibitors** | | | |
| Dark circles under eyes | 24.26 | 0.47 | 20 |
| Eye disorder | 48.55 | 0.46 | 123 |
| Cataract | 67.12 | 0.44 | 239 |
| Blindness | 40.61 | 0.41 | 122 |
| Visual impairment | 73.25 | 0.35 | 442 |
| Glaucoma | 18.10 | 0.28 | 44 |
| Myopia | 15.27 | 0.26 | 21 |
| Dry eye | 28.54 | 0.22 | 238 |
| Eyelid disorder | 12.04 | 0.15 | 13 |
| Eye pruritus | 12.55 | 0.14 | 79 |
| Eye allergy | 11.71 | 0.13 | 12 |
| Lacrimation increased | 15.50 | 0.11 | 295 |
| Vision blurred | 13.00 | 0.08 | 334 |
| **Palbociclib** | | | |
| Cataract | 100.83 | 0.64 | 207 |
| Blindness | 40.08 | 0.48 | 93 |
| Eye disorder | 32.42 | 0.42 | 83 |
| Dry eye | 29.18 | 0.28 | 180 |
| Visual impairment | 31.15 | 0.24 | 282 |
| Lacrimation increased | 20.09 | 0.18 | 228 |
| Eye pruritus | 10.98 | 0.14 | 58 |
| Glaucoma | 9.98 | 0.13 | 28 |
| Eye discharge | 9.43 | 0.12 | 30 |
| Eye allergy | 11.07 | 0.10 | 9 |
| Vision blurred | 8.75 | 0.05 | 237 |
| **Ribociclib** | | | |
| Myopia | 114.79 | 1.56 | 17 |
| Dark circles under eyes | 116.51 | 1.53 | 14 |
| Visual impairment | 189.76 | 1.19 | 148 |
| Eye disorder | 70.95 | 1.11 | 38 |
| Eyelid disorder | 51.99 | 0.88 | 8 |
| Eye inflammation | 34.59 | 0.80 | 11 |
| Glaucoma | 32.92 | 0.79 | 15 |
| Eye swelling | 27.41 | 0.63 | 27 |
| Eyelid margin crusting | 31.84 | 0.49 | 6 |
| Blindness | 19.95 | 0.48 | 25 |
| Eye pruritus | 16.90 | 0.42 | 21 |
| Dry eye | 19.75 | 0.39 | 51 |
| Vision blurred | 21.06 | 0.35 | 78 |
| Eye colour change | 37.14 | 0.34 | 5 |
| Photopsia | 12.17 | 0.23 | 9 |
| Diplopia | 8.38 | 0.13 | 20 |
| Astigmatism | 12.97 | 0.03 | 5 |

*Abemaciclib has no positive signal.
