# Supplementary material for: Ocular toxicity events of cyclin-dependent kinase 4/6 inhibitors in breast cancer: a pharmacovigilance study based on the faers database
Source: Front Pharmacol. 2025 Nov 6;16:1668446. doi: 10.3389/fphar.2025.1668446 (PMC12631214; doi:10.3389/fphar.2025.1668446)
Supplement: Supplementary file 4 [file Table3.docx]

**Table S3** Results of adverse events related to ocular toxicity formed by PRR method

| PT | PRR | PRR_CI_up | PRR_CI_down | Cases |
| --- | --- | --- | --- | --- |
| **CDK4/6 inhibitors** | | | | |
| Visual impairment | 1.67 | 1.89 | 1.49 | 442 |
| Vision blurred | 1.27 | 1.45 | 1.12 | 334 |
| Lacrimation increased | 1.32 | 1.52 | 1.15 | 295 |
| Cataract | 2.00 | 2.36 | 1.69 | 239 |
| Dry eye | 1.54 | 1.81 | 1.31 | 238 |
| Eye disorder | 2.32 | 2.95 | 1.82 | 123 |
| Blindness | 2.14 | 2.72 | 1.68 | 122 |
| Eye pruritus | 1.66 | 2.20 | 1.25 | 79 |
| Eye pain | 1.15 | 1.52 | 0.87 | 71 |
| Eye swelling | 1.06 | 1.44 | 0.78 | 58 |
| Diplopia | 0.83 | 1.15 | 0.60 | 49 |
| Glaucoma | 2.37 | 3.56 | 1.57 | 44 |
| Eye irritation | 0.74 | 1.05 | 0.53 | 41 |
| Ocular hyperaemia | 0.63 | 0.89 | 0.44 | 39 |
| Eye discharge | 1.72 | 2.62 | 1.13 | 36 |
| Eye haemorrhage | 1.24 | 1.97 | 0.79 | 27 |
| Visual acuity reduced | 0.36 | 0.55 | 0.23 | 24 |
| Blindness unilateral | 1.56 | 2.62 | 0.93 | 23 |
| Myopia | 3.39 | 6.49 | 1.77 | 21 |
| Dark circles under eyes | 5.73 | 12.59 | 2.61 | 20 |
| Ocular discomfort | 1.36 | 2.37 | 0.78 | 19 |
| Photophobia | 0.42 | 0.67 | 0.26 | 19 |
| Macular degeneration | 0.91 | 1.56 | 0.53 | 18 |
| Photopsia | 1.16 | 2.02 | 0.67 | 18 |
| Vitreous floaters | 1.18 | 2.13 | 0.65 | 16 |
| Eye inflammation | 1.59 | 2.96 | 0.85 | 16 |
| Eye movement disorder | 1.34 | 2.55 | 0.70 | 14 |
| Asthenopia | 0.59 | 1.07 | 0.32 | 13 |
| Eyelid disorder | 4.19 | 10.12 | 1.74 | 13 |
| Eye allergy | 4.42 | 11.23 | 1.74 | 12 |
| Keratitis | 0.52 | 0.99 | 0.27 | 11 |
| Retinal detachment | 0.25 | 0.47 | 0.14 | 11 |
| Eyelid margin crusting | 3.55 | 8.82 | 1.43 | 11 |
| Periorbital swelling | 1.58 | 3.34 | 0.74 | 11 |
| Retinal haemorrhage | 0.83 | 1.70 | 0.41 | 10 |
| Eyelid ptosis | 0.76 | 1.54 | 0.37 | 10 |
| Optic nerve disorder | 1.98 | 4.53 | 0.87 | 10 |
| Swelling of eyelid | 0.74 | 1.49 | 0.37 | 10 |
| Visual field defect | 0.44 | 0.93 | 0.21 | 8 |
| Conjunctival haemorrhage | 0.69 | 1.50 | 0.32 | 8 |
| Eyelids pruritus | 2.58 | 6.87 | 0.97 | 8 |
| Uveitis | 0.32 | 0.66 | 0.15 | 8 |
| Maculopathy | 0.43 | 0.91 | 0.20 | 8 |
| Eye colour change | 5.16 | 17.14 | 1.55 | 8 |
| Dacryostenosis acquired | 0.14 | 0.29 | 0.06 | 7 |
| Eye oedema | 0.72 | 1.67 | 0.31 | 7 |
| Ocular vascular disorder | 2.01 | 5.39 | 0.75 | 7 |
| Diabetic retinopathy | 3.10 | 10.14 | 0.94 | 6 |
| Macular oedema | 0.27 | 0.62 | 0.12 | 6 |
| Strabismus | 1.43 | 4.28 | 0.48 | 5 |
| Astigmatism | 0.92 | 2.56 | 0.33 | 5 |
| Blindness transient | 0.72 | 1.93 | 0.27 | 5 |
| Retinal vein occlusion | 0.72 | 1.93 | 0.27 | 5 |
| Papilloedema | 0.25 | 0.62 | 0.10 | 5 |
| Xerophthalmia | 0.52 | 1.35 | 0.20 | 5 |
| Abnormal sensation in eye | 0.61 | 1.80 | 0.20 | 4 |
| Blepharitis | 0.37 | 1.05 | 0.13 | 4 |
| Ulcerative keratitis | 0.34 | 0.98 | 0.12 | 4 |
| Periorbital oedema | 0.23 | 0.64 | 0.08 | 4 |
| Foreign body sensation in eyes | 0.24 | 0.67 | 0.09 | 4 |
| Cornea verticillata | 1.03 | 3.29 | 0.32 | 4 |
| Mydriasis | 0.31 | 1.03 | 0.09 | 3 |
| Eyelid oedema | 0.11 | 0.34 | 0.03 | 3 |
| Erythema of eyelid | 0.65 | 2.29 | 0.18 | 3 |
| Optic ischaemic neuropathy | 2.58 | 12.78 | 0.52 | 3 |
| Vitreous detachment | 0.22 | 0.70 | 0.07 | 3 |
| **Palbociclib** | | | | |
| Visual impairment | 1.48 | 1.70 | 1.29 | 282 |
| Vision blurred | 1.25 | 1.45 | 1.08 | 237 |
| Lacrimation increased | 1.42 | 1.65 | 1.22 | 228 |
| Cataract | 2.39 | 2.85 | 2.01 | 207 |
| Dry eye | 1.62 | 1.93 | 1.36 | 180 |
| Eye disorder | 2.16 | 2.84 | 1.65 | 83 |
| Blindness | 2.26 | 2.93 | 1.74 | 93 |
| Eye pruritus | 1.68 | 2.30 | 1.23 | 58 |
| Eye pain | 1.15 | 1.57 | 0.84 | 51 |
| Eye swelling | 0.73 | 1.10 | 0.49 | 29 |
| Diplopia | 0.61 | 0.93 | 0.40 | 26 |
| Glaucoma | 2.08 | 3.32 | 1.31 | 28 |
| Eye irritation | 0.73 | 1.09 | 0.49 | 29 |
| Ocular hyperaemia | 0.62 | 0.93 | 0.42 | 28 |
| Eye discharge | 1.98 | 3.10 | 1.27 | 30 |
| Eye haemorrhage | 1.53 | 2.47 | 0.95 | 24 |
| Visual acuity reduced | 0.45 | 0.70 | 0.29 | 22 |
| Blindness unilateral | 1.60 | 2.83 | 0.90 | 17 |
| Myopia | 0.89 | 2.67 | 0.30 | 4 |
| Dark circles under eyes | 2.38 | 6.69 | 0.85 | 6 |
| Ocular discomfort | 1.29 | 2.43 | 0.68 | 13 |
| Photophobia | 0.42 | 0.74 | 0.24 | 14 |
| Macular degeneration | 1.19 | 2.06 | 0.69 | 17 |
| Photopsia | 0.80 | 1.66 | 0.39 | 9 |
| Vitreous floaters | 1.33 | 2.51 | 0.70 | 13 |
| Eye inflammation | 0.69 | 1.79 | 0.26 | 5 |
| Eye movement disorder | 1.32 | 2.73 | 0.64 | 10 |
| Asthenopia | 0.81 | 1.49 | 0.45 | 13 |
| Eyelid disorder | 2.23 | 6.83 | 0.73 | 5 |
| Eye allergy | 4.59 | 12.33 | 1.71 | 9 |
| Keratitis | 0.13 | 0.53 | 0.03 | 2 |
| Retinal detachment | 0.16 | 0.39 | 0.06 | 5 |
| Eyelid margin crusting | 1.79 | 5.93 | 0.54 | 4 |
| Periorbital swelling | 1.79 | 3.98 | 0.80 | 9 |
| Retinal haemorrhage | 0.92 | 2.01 | 0.42 | 8 |
| Eyelid ptosis | 0.84 | 1.82 | 0.39 | 8 |
| Optic nerve disorder | 1.65 | 4.34 | 0.63 | 6 |
| Swelling of eyelid | 0.31 | 1.00 | 0.09 | 3 |
| Visual field defect | 0.46 | 1.07 | 0.20 | 6 |
| Conjunctival haemorrhage | 0.95 | 2.08 | 0.44 | 8 |
| Eyelids pruritus | 3.13 | 8.62 | 1.13 | 7 |
| Uveitis | 0.27 | 0.68 | 0.11 | 5 |
| Maculopathy | 0.60 | 1.26 | 0.28 | 8 |
| Eye colour change | 2.68 | 11.97 | 0.60 | 3 |
| Dacryostenosis acquired | 0.16 | 0.37 | 0.07 | 6 |
| Eye oedema | 0.57 | 1.64 | 0.20 | 4 |
| Ocular vascular disorder | 2.38 | 6.69 | 0.85 | 6 |
| Diabetic retinopathy | 3.57 | 12.34 | 1.03 | 5 |
| Macular oedema | 0.31 | 0.77 | 0.12 | 5 |
| Blindness transient | 0.79 | 2.35 | 0.27 | 4 |
| Retinal vein occlusion | 0.60 | 2.02 | 0.18 | 3 |
| Papilloedema | 0.27 | 0.76 | 0.10 | 4 |
| Xerophthalmia | 0.43 | 1.42 | 0.13 | 3 |
| Abnormal sensation in eye | 0.42 | 1.82 | 0.10 | 2 |
| Blepharitis | 0.26 | 1.07 | 0.06 | 2 |
| Ulcerative keratitis | 0.24 | 1.00 | 0.06 | 2 |
| Periorbital oedema | 0.32 | 0.88 | 0.11 | 4 |
| Foreign body sensation in eyes | 0.33 | 0.93 | 0.12 | 4 |
| Cornea verticillata | 0.36 | 2.79 | 0.05 | 1 |
| Eyelid oedema | 0.15 | 0.47 | 0.05 | 3 |
| Erythema of eyelid | 0.89 | 3.17 | 0.25 | 3 |
| Optic ischaemic neuropathy | 2.38 | 14.25 | 0.40 | 2 |
| Vitreous detachment | 0.10 | 0.72 | 0.01 | 1 |
| **Ribociclib** | | | | |
| Visual impairment | 3.24 | 3.87 | 2.72 | 148 |
| Vision blurred | 1.72 | 2.17 | 1.36 | 78 |
| Lacrimation increased | 1.25 | 1.67 | 0.93 | 48 |
| Cataract | 1.45 | 2.11 | 1.00 | 30 |
| Dry eye | 1.91 | 2.56 | 1.43 | 51 |
| Eye disorder | 4.14 | 5.93 | 2.89 | 38 |
| Blindness | 2.54 | 3.88 | 1.66 | 25 |
| Eye pruritus | 2.55 | 4.05 | 1.61 | 21 |
| Eye pain | 1.69 | 2.75 | 1.04 | 18 |
| Eye swelling | 2.86 | 4.31 | 1.89 | 27 |
| Diplopia | 1.96 | 3.13 | 1.23 | 20 |
| Glaucoma | 4.67 | 8.33 | 2.61 | 15 |
| Eye irritation | 1.05 | 2.00 | 0.55 | 10 |
| Ocular hyperaemia | 0.93 | 1.76 | 0.49 | 10 |
| Eye discharge | 0.83 | 2.65 | 0.26 | 3 |
| Eye haemorrhage | 0.80 | 2.55 | 0.25 | 3 |
| Visual acuity reduced | 0.17 | 0.69 | 0.04 | 2 |
| Blindness unilateral | 2.36 | 5.57 | 1.00 | 6 |
| Myopia | 15.86 | 31.38 | 8.02 | 17 |
| Dark circles under eyes | 23.22 | 53.64 | 10.05 | 14 |
| Ocular discomfort | 2.49 | 5.90 | 1.05 | 6 |
| Photophobia | 0.63 | 1.55 | 0.26 | 5 |
| Macular degeneration | 0.29 | 2.12 | 0.04 | 1 |
| Photopsia | 3.36 | 6.92 | 1.63 | 9 |
| Vitreous floaters | 1.28 | 4.16 | 0.39 | 3 |
| Eye inflammation | 6.32 | 12.78 | 3.12 | 11 |
| Eye movement disorder | 1.66 | 5.47 | 0.50 | 3 |
| Eyelid disorder | 14.93 | 39.77 | 5.60 | 8 |
| Eye allergy | 6.40 | 24.74 | 1.65 | 3 |
| Keratitis | 2.44 | 4.94 | 1.21 | 9 |
| Retinal detachment | 0.66 | 1.62 | 0.27 | 5 |
| Eyelid margin crusting | 11.20 | 32.26 | 3.89 | 6 |
| Periorbital swelling | 0.83 | 6.21 | 0.11 | 1 |
| Retinal haemorrhage | 0.96 | 4.02 | 0.23 | 2 |
| Eyelid ptosis | 0.44 | 3.21 | 0.06 | 1 |
| Optic nerve disorder | 4.59 | 14.08 | 1.50 | 4 |
| Swelling of eyelid | 1.71 | 4.80 | 0.61 | 4 |
| Visual field defect | 0.64 | 2.61 | 0.15 | 2 |
| Uveitis | 0.23 | 1.65 | 0.03 | 1 |
| Eye colour change | 18.66 | 69.48 | 5.01 | 5 |
| Dacryostenosis acquired | 0.11 | 0.81 | 0.02 | 1 |
| Eye oedema | 1.79 | 5.93 | 0.54 | 3 |
| Ocular vascular disorder | 1.66 | 13.09 | 0.21 | 1 |
| Diabetic retinopathy | 2.99 | 25.55 | 0.35 | 1 |
| Strabismus | 6.63 | 21.54 | 2.04 | 4 |
| Astigmatism | 5.33 | 14.80 | 1.92 | 5 |
| Papilloedema | 0.29 | 2.08 | 0.04 | 1 |
| Xerophthalmia | 1.19 | 5.04 | 0.28 | 2 |
| Abnormal sensation in eye | 1.76 | 7.60 | 0.41 | 2 |
| Blepharitis | 0.53 | 3.92 | 0.07 | 1 |
| Ulcerative keratitis | 1.00 | 4.16 | 0.24 | 2 |
| Cornea verticillata | 4.48 | 16.27 | 1.23 | 3 |
| Mydriasis | 1.79 | 5.93 | 0.54 | 3 |
| Vitreous detachment | 0.41 | 3.02 | 0.06 | 1 |
| **Abemaciclib** | | | | |
| Visual impairment | 0.43 | 0.77 | 0.24 | 12 |
| Vision blurred | 0.69 | 1.09 | 0.44 | 19 |
| Lacrimation increased | 0.81 | 1.28 | 0.51 | 19 |
| Cataract | 0.16 | 0.64 | 0.04 | 2 |
| Dry eye | 0.43 | 0.91 | 0.20 | 7 |
| Eye disorder | 0.36 | 1.45 | 0.09 | 2 |
| Blindness | 0.67 | 1.81 | 0.25 | 4 |
| Eye pain | 0.31 | 1.25 | 0.08 | 2 |
| Eye swelling | 0.35 | 1.41 | 0.09 | 2 |
| Diplopia | 0.49 | 1.52 | 0.15 | 3 |
| Glaucoma | 0.51 | 3.71 | 0.07 | 1 |
| Eye irritation | 0.35 | 1.40 | 0.09 | 2 |
| Ocular hyperaemia | 0.15 | 1.09 | 0.02 | 1 |
| Eye discharge | 1.37 | 4.37 | 0.43 | 3 |
| Eye movement disorder | 0.91 | 6.70 | 0.12 | 1 |
| Retinal detachment | 0.22 | 1.56 | 0.03 | 1 |
| Eyelid margin crusting | 3.07 | 24.56 | 0.38 | 1 |
| Periorbital swelling | 1.37 | 10.23 | 0.18 | 1 |
| Eyelid ptosis | 0.72 | 5.28 | 0.10 | 1 |
| Swelling of eyelid | 2.11 | 6.85 | 0.65 | 3 |
| Eyelids pruritus | 3.07 | 24.56 | 0.38 | 1 |
| Uveitis | 0.76 | 3.09 | 0.19 | 2 |
| Macular oedema | 0.42 | 3.06 | 0.06 | 1 |
| Strabismus | 2.73 | 21.55 | 0.35 | 1 |
| Blindness transient | 1.37 | 10.23 | 0.18 | 1 |
| Retinal vein occlusion | 2.73 | 11.77 | 0.63 | 2 |
| Blepharitis | 0.88 | 6.45 | 0.12 | 1 |
| Optic ischaemic neuropathy | 8.19 | 78.75 | 0.85 | 1 |
| Vitreous detachment | 0.68 | 4.98 | 0.09 | 1 |
