# Supplementary material for: Ocular toxicity events of cyclin-dependent kinase 4/6 inhibitors in breast cancer: a pharmacovigilance study based on the faers database
Source: Front Pharmacol. 2025 Nov 6;16:1668446. doi: 10.3389/fphar.2025.1668446 (PMC12631214; doi:10.3389/fphar.2025.1668446)
Supplement: Supplementary file 5 [file Table4.docx]

**Table S4 Serious Proportion and Death Proportion of Ocular Toxicity Events Reported by CDK4/6 Inhibitors of Top20**

|  | PT | Cases | Total_Cases | Serious_Cases | Death_Cases | Serious_Proportion（%） | Death_Proportion（%） |
| --- | --- | --- | --- | --- | --- | --- | --- |
| 1 | Visual impairment | 442 | 442 | 64 | 29 | 14.48 | 6.56 |
| 2 | Vision blurred | 334 | 334 | 37 | 20 | 11.08 | 5.99 |
| 3 | Lacrimation increased | 295 | 295 | 8 | 16 | 2.71 | 5.42 |
| 4 | Cataract | 239 | 239 | 6 | 13 | 2.51 | 5.44 |
| 5 | Dry eye | 238 | 238 | 16 | 14 | 6.72 | 5.88 |
| 6 | Eye disorder | 123 | 123 | 24 | 9 | 19.51 | 7.32 |
| 7 | Blindness | 122 | 122 | 12 | 10 | 9.84 | 8.20 |
| 8 | Eye pruritus | 79 | 79 | 4 | 8 | 5.06 | 10.13 |
| 9 | Eye pain | 71 | 71 | 12 | 5 | 16.90 | 7.04 |
| 10 | Eye swelling | 58 | 58 | 14 | 4 | 24.14 | 6.90 |
| 11 | Diplopia | 49 | 49 | 10 | 0 | 20.41 | 0.00 |
| 12 | Glaucoma | 44 | 44 | 4 | 6 | 9.09 | 13.64 |
| 13 | Eye irritation | 41 | 41 | 2 | 2 | 4.88 | 4.88 |
| 14 | Ocular hyperaemia | 39 | 39 | 0 | 1 | 0.00 | 2.56 |
| 15 | Eye discharge | 36 | 36 | 0 | 2 | 0.00 | 5.56 |
| 16 | Eye haemorrhage | 27 | 27 | 0 | 0 | 0.00 | 0.00 |
| 17 | Visual acuity reduced | 24 | 24 | 4 | 0 | 16.67 | 0.00 |
| 18 | Blindness unilateral | 23 | 23 | 2 | 2 | 8.70 | 8.70 |
| 19 | Myopia | 21 | 21 | 12 | 2 | 57.14 | 9.52 |
| 20 | Dark circles under eyes | 20 | 20 | 8 | 2 | 40.00 | 10.00 |
